# A combined microbial and biogeochemical dataset from high-latitude ecosystems with respect to methane cycle

**DOI:** 10.1038/s41597-022-01759-8

**Published:** 2022-11-04

**Authors:** Maialen Barret, Laure Gandois, Frederic Thalasso, Karla Martinez Cruz, Armando Sepulveda Jauregui, Céline Lavergne, Roman Teisserenc, Polette Aguilar, Oscar Gerardo Nieto, Claudia Etchebehere, Bruna Martins Dellagnezze, Patricia Bovio Winkler, Gilberto J. Fochesatto, Nikita Tananaev, Mette M. Svenning, Christophe Seppey, Alexander Tveit, Rolando Chamy, María Soledad Astorga España, Andrés Mansilla, Anton Van de Putte, Maxime Sweetlove, Alison E. Murray, Léa Cabrol

**Affiliations:** 1 Laboratoire d’Ecologie Fonctionnelle et Environnement, Université de Toulouse, CNRS, Toulouse, France; 2 Biotechnology and Bioengineering Department, Center for Research and Advanced Studies (Cinvestav), Mexico City, Mexico; 3 University of Magallanes, Punta Arenas, Chile; 4 Environmental Physics Group, Limnological Institute, University of Konstanz, Konstanz, Germany; 5 Center for Climate and Resilience Research (CR)2, Santiago, Chile; 6 HUB AMBIENTAL UPLA, Universidad Playa Ancha, Valparaíso, Chile; 7 Unidad Académica de Ecología y Biodiversidad Acuática, Instituto de Ciencias del Mar y Limnología, UNAM, Mexico City, México; 8 Microbial Ecology Laboratory, BioGem Department, Biological Research Institute Clemente Estable, Montevideo, Uruguay; 9 Department of Atmospheric Sciences, University of Alaska Fairbanks, Fairbanks, Alaska USA; 10 Melnikov Permafrost Institute, Yakutsk, Russia; 11 North-Eastern Federal University, Yakutsk, Russia; 12 Department of Arctic and Marine Biology, UiT The Arctic University of Norway, Tromsoe, Norway; 13 Institute of Environmental Science and Geography, University of Potsdam, Potsdam, Germany; 14 Pontifical Catholic University of Valparaíso, Valparaiso, Chile; 15 BEDIC, OD Nature, Royal Belgian Institute of Natural Sciences, Brussels, Belgium; 16 Division of Earth and Ecosystem sciences, Desert Research Institute, Reno, NV USA; 17 Aix-Marseille University, Univ Toulon, CNRS, IRD, M.I.O. UM 110, Mediterranean Institute of Oceanography, Marseille, France; 18 Millenium Institute “Biodiversity of Antartic and Subantarctic Ecosystems” (BASE), Santiago, Chile

**Keywords:** Climate-change ecology, Microbial ecology, Carbon cycle

## Abstract

High latitudes are experiencing intense ecosystem changes with climate warming. The underlying methane (CH_4_) cycling dynamics remain unresolved, despite its crucial climatic feedback. Atmospheric CH_4_ emissions are heterogeneous, resulting from local geochemical drivers, global climatic factors, and microbial production/consumption balance. Holistic studies are mandatory to capture CH_4_ cycling complexity. Here, we report a large set of integrated microbial and biogeochemical data from 387 samples, using a concerted sampling strategy and experimental protocols. The study followed international standards to ensure inter-comparisons of data amongst three high-latitude regions: Alaska, Siberia, and Patagonia. The dataset encompasses different representative environmental features (e.g. lake, wetland, tundra, forest soil) of these high-latitude sites and their respective heterogeneity (e.g. characteristic microtopographic patterns). The data included physicochemical parameters, greenhouse gas concentrations and emissions, organic matter characterization, trace elements and nutrients, isotopes, microbial quantification and composition. This dataset addresses the need for a robust physicochemical framework to conduct and contextualize future research on the interactions between climate change, biogeochemical cycles and microbial communities at high-latitudes.

## Abstract (summary)

**Table:** 

Measurement(s)	microbial diversity • microbial abundances • cations and anions • trace elements
Technology Type(s)	MiSeq sequencing 16S rRNA gene • Real Time PCR • HPLC • ICP-MS
Sample Characteristic - Organism	bacteria • archaea
Sample Characteristic - Environment	lake water • lake sediment • wetland • peatland • soil • pond
Sample Characteristic - Location	Western Siberia • Alaska • Patagonia • Cape Horn province • Magellanic subantarctic ecoregion

## Background & Summary

Almost half of worldwide CH_4_ emissions originate from natural ecosystems, among which aquatic ecosystems, i.e. wetlands and other inland waters, are major contributors^1^. At high latitudes, where the highest density of freshwater ecosystems is found^2^, extreme variability is occurring as a result of climate change^3^. In the Arctic, air temperatures are expected to rise twice as fast as the global average^4^. Higher air temperature speeds up permafrost thawing, making available a larger amount of sequestered organic matter for microbial degradation and mineralisation. Permafrost thawing can also lead to anoxic conditions in the resulting soil, peat and/or water bodies, potentially enhancing CH_4_ emissions. Any disturbance of natural CH_4_ cycle can constitute a strong positive feedback on global climate, considering the strong radiative effect of this greenhouse gas^5,6^. For these reasons, documenting microbially-mediated CH_4_ emissions in high-latitude ecosystems is essential to determine tipping points on the positive feedback to climate warming. Climatic projections may also impact significantly the CH_4_ emissions from Sub-Antarctic environments. Ecosystems in the Magellanic ecoregion^7^ have been understudied compared to their northern counterparts^8^ and CH_4_ cycling has been rarely investigated^9,10^. However, this region is of major importance since it is an expansive, unique continental area between 45 and 55 °S, it is very sparsely populated and the ecology of the region has been highly conserved (Cape Horn Biosphere Reserve, UNESCO).

Net atmospheric CH_4_ emissions from terrestrial and aquatic ecosystems reflect the balance between CH_4_ production, transport, and oxidation in these ecosystems. Methane is primarily produced by methanogenic Archaea through anaerobic decomposition of organic matter. Methanogenesis can occur via the acetoclastic, hydrogenotrophic or methylotrophic pathways, the contribution of each depending on temperature, substrate availability, and microbial interactions^11^. This being the only biological CH_4_ source has been challenged by recent evidences for CH_4_ production in oxic conditions^12,13^, contributing for about 20% of CH_4_ emissions from lakes^14,15^. However, the global significance of oxic CH_4_ production, its underlying metabolic pathways, and the identity of microorganisms actively involved in this process are not fully constrained yet^16^. Methane emissions are strongly mitigated by microbial oxidation. For example, in aquatic ecosystems, 51–100% of the CH_4_ produced in deep sediments can be oxidized in the water column, before reaching the atmosphere^17,18^. Methane oxidation is carried out in oxic conditions by aerobic methane-oxidizing bacteria (MOB) belonging to Alphaproteobacteria, Gammaproteobacteria, and Verrucomicrobia^19^. On the other hand, anaerobic oxidation of methane (AOM) has been also identified as a major process in aquatic^20,21^ and terrestrial^22,23^ ecosystems, and attributed to anaerobic methane-oxidizing Archaea (ANME)^24^, bacteria from the NC10 phylum^25^ or Gammaproteobacteria MOB active in anoxic zones^20,26^.

At the landscape scale, these processes occur with different magnitude depending on ecosystem type and associated microtopography. Soils are primarily CH_4_ sinks due to the drawdown of even very low levels of atmospheric CH_4_^27,28^. Lakes and wetlands are recognized as major CH_4_ emission sources, despite high flux variability between and within aquatic ecosystems^1,29^. Among wetlands, organic-rich peatlands are estimated to cover more than 3.7 × 10^6^ km^2^ in northern high latitudes^30^ and to emit 30 Tg CH_4_ yr^−1^ ^31^. These important CH_4_ contributors are complex ecosystems with a variety of hydrologic regimes, productivity levels, vegetation covers, and variability in CH_4_ emissions. Especially, lower emissions are generally observed in fens compared to bogs, in association with different CH_4_ production pathways^32^. Temperature, water-table level, and permafrost state also influence CH_4_ emissions^33^. In permafrost landscapes, CH_4_ emissions from ‘wet features’, characteristic of degrading permafrost (e.g. ponds, hollows, thaw lakes, internal lawns, and collapse scars), are usually higher than from ‘dry features’, characteristic of intact permafrost (e.g. pingos, polygonal peatlands, hummocks, palsa, or peat plateau)^34^. Physicochemical characteristics such as pH, nitrogen, and phosphorus availability, and carbon source quality and quantity also drive CH_4_ emissions^35–37^. In the context of global warming, the expected variations of geochemical and physicochemical factors may affect the microbial CH_4_-cycle.

The present study focuses on the CH_4_ cycle in three high-latitude regions (Alaska, Patagonia, Siberia). Three ecosystem types (soils, wetlands, and lakes) have been investigated in each region during summer, with a systematic evaluation of their different habitats. This data set addresses the recent call of the global community of microbial scientists for the integration of microorganisms in mainstream climate change research addressing carbon fluxes^38,39^. Here, a thorough analysis of microbial community diversity and structure, carried out through functional gene quantifications and high throughput 16S rRNA gene amplicon sequencing, has been coupled with the physicochemical characterisation of habitats and measurement of atmospheric CH_4_ and CO_2_ fluxes. The physicochemical analysis included quantification of nutrients and trace elements, as well as stable isotopic signature of carbon species (CH_4_, dissolved organic and inorganic carbon) to track CH_4_ production and oxidation pathways. This database offers the possibility to expand the geographical scope of microbial ecology, biogeographic, and/or biogeochemical studies (either related to C cycling or other cycles) towards high latitude ecosystems. Moreover, this database is of particular interest for the earth system science community in order to parameterize relevant surface and sub-surface biogeochemical processes that can be further used to refine climate models or global models.

## Methods

### Sites overview and characteristics

**Fig. 1:**
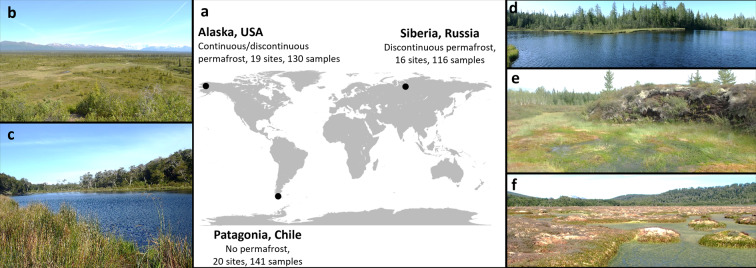
Location of the three areas included in this study (panel **a**). The permafrost state and the number of sites and samples per region is indicated for each area. General views of 5 sites are provided as examples (**b**–**f**). Panel B provides a large view of the ecosystem surrounding the wetland ALP2 (Alaska, exact location indicated by the white circle). Lake PCL1 (panel **c**) is representative of the lakes on Navarino island (Chilean Patagonia). The glacial lake SIL2 is shown in panel d. At site SIP5, the hollow at first plan is surrounded by palsa (hummock, second plan), characterized by dark organic matter and lichen vegetation (panel **e**). The PPP3 peatland shown in panel f is dominated by *Sphagnum magellanicum*, like most peatlands in the area.

**Fig. 2:**
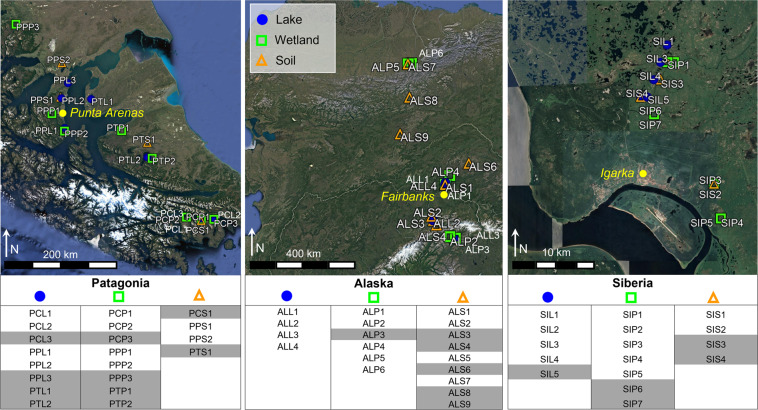
Maps of sampling sites in Patagonia, Alaska and Siberia, indicating the ecosystem type (lake, wetland, soil). The tables show the complete- (in white) and the partial- (in grey) characterization sites. The exact coordinates of each sample are provided in the data record (See data records section).

This study focused on three regions located in subantarctic, arctic, and subarctic latitudes. The respective latitudinal and longitudinal ranges covered in this study were: 54.95 to 52.08 °S, and 72.03 to 67.34 °W in Patagonia; 67.44 to 67.54 °N, and 86.59 to 86.71 °E in Siberia; 63.21 to 68.63 °N, and −150.79 to −145.98 °W in Alaska (Figs. 1 and 2). The exact coordinates for each sample were included in the submitted dataset. The field campaigns were conducted in 2016, during the summer for each respective region: January-February in Chilean Patagonia, June-July in Alaska and July-August in Siberia.

**Fig. 3:**
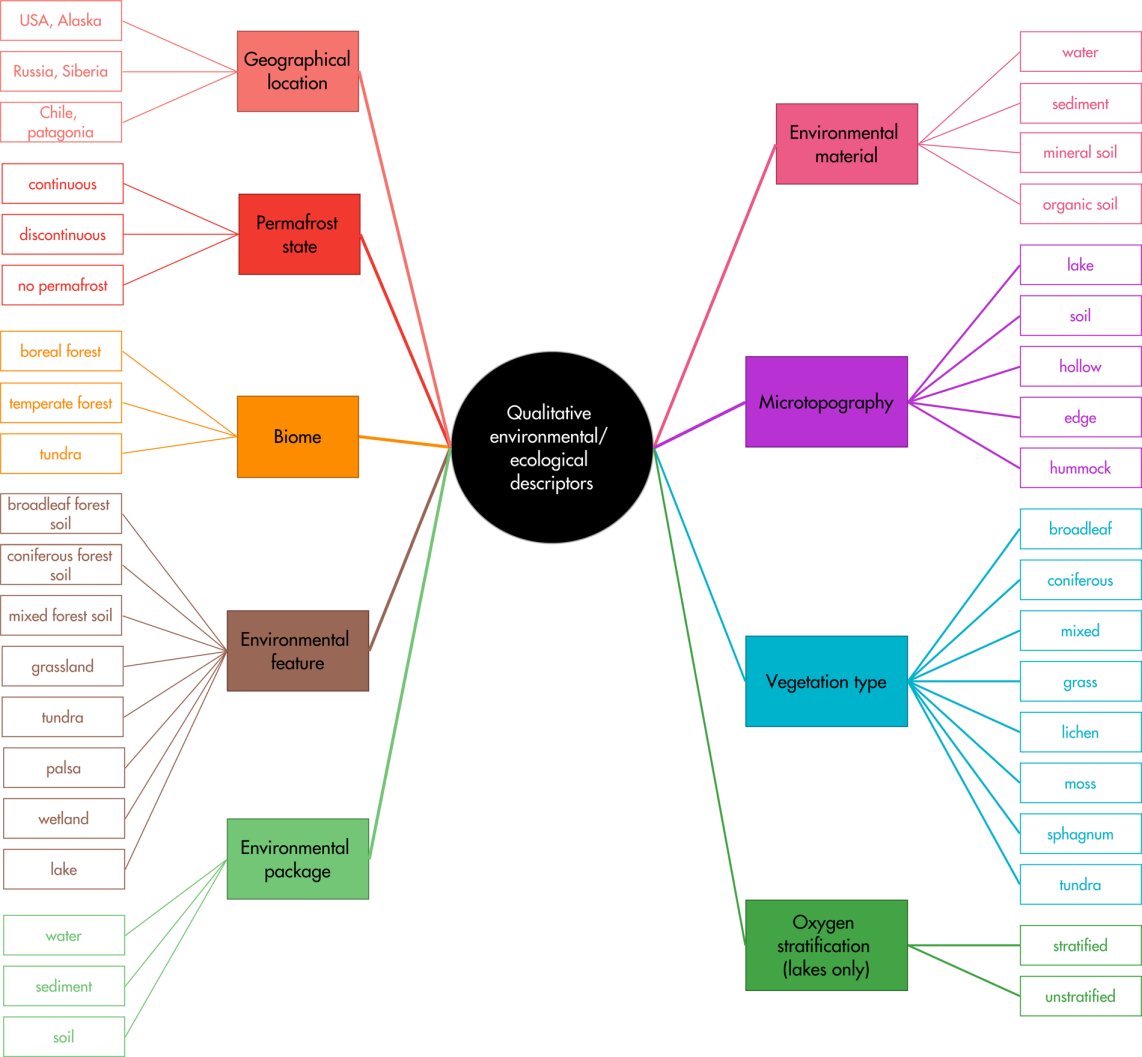
Description of the qualitative environmental/ecological descriptors used to describe every sample, derived from ENVO Environment Ontology^40^.

For every site included in the present study, a set of nine qualitative environmental and/or ecological site-scale descriptors was selected and adapted from ENVO Environment Ontology^40^, which included for example permafrost state, biome, environmental feature and vegetation type (Table 1, Fig. 3). Permafrost state was obtained from the NSIDC permafrost map^41^. The biome, large-scale descriptor based on climate and vegetation criteria, was derived from Olson *et al*.^42^. Temperate forest, boreal forest, and tundra biomes were included. The environmental features that were representative for the three regions were considered: lakes, wetlands, broadleaf/coniferous/mixed forest soils, grassland, tundra, and palsa. All the metadata was included in the submitted dataset. Table 2 summarizes the main types of sampled ecosystems and their main characteristics in the three regions, while Supplementary Table S1 provides the details of each sampling site.

**Table 1:** Overview of the dataset contained in Mimarks sheet.

Ecological/environmental descriptors	Bioclimatic variables	Physicochemical characteristics
**Site-scale descriptors:** Region* Biome* Elevation* Environmental feature* Geographical location* Oxygen stratification* Permafrost state* Total depth of water column **Point-scale descriptors:** Latitude* Longitude* Microtopography* Vegetation type* **Sample-scale descriptors:** Environmental material* Environmental package* Sample depth*	Annual Mean Temperature* Annual Precipitation* Isothermality* Max Temperature of Warmest Month* Mean Diurnal Range* Mean Temperature of Coldest Quarter* Mean Temperature of Driest Quarter* Mean Temperature of Warmest Quarter* Mean Temperature of Wettest Quarter* Min Temperature of Coldest Month * Precipitation of Coldest Quarter* Precipitation of Driest Month* Precipitation of Driest Quarter* Precipitation of Warmest Quarter* Precipitation of Wettest Month* Precipitation of Wettest Quarter* Precipitation Seasonality* Temperature Annual Range* Temperature Seasonality*	Conductivity* δ^13^C-DOC Dissolved organic carbon Dissolved oxygen Dry weight* Organic matter pH* Redox potential Suspended organic matter Suspended particulate matter Temperature* Total organic carbon Volatile solids percentage Water content **Anions and cations:** ammonium, bromide, calcium, chloride, magnesium, nitrate, nitrite, phosphate, potassium, sodium, fluoride, sulphate **Trace elements:** aluminium, antimony, arsenic, barium, cadmium, caesium, chromium, cobalt, copper, iron, lanthanium, lead, manganese, nickel, rubidium, strontium, titanium, uranium, vanadium, zinc **Optical properties:** Absorbance et 254 nm, Specific UV absorbance, Fluorescence index
**GHG cycling**	**Microbial variables**	
CH_4_ emission rate CO_2_ emission rate Dissolved methane Dissolved inorganic carbon δ^2^H-CH_4_ δ^13^C-CH_4_ δ^13^C-CO_2_	Archaeal abundance* Bacterial abundance* Methanogen abundance* Methanotroph abundance* Microbial community composition*	
		**Miscellaneous**
		Characterization type* Collection date* Ecosystem nomenclature* Official ecosystem name*

Units are provided for each variable in the database. Data provided for every sample are indicated with the symbol *, while others are available only for sites with complete characterization (full symbols), or for certain environmental features or packages (soil and sediment samples in brown; water samples in blue). Empty symbols represent data available for partial-characterization sites.

**Table 2:** Main types of sampled ecosystems in the three studied regions.

	Patagonia	Alaska	Siberia
Soils	- Broadleaf forest (*Nothofagus*)	- From mixed boreal forest to taiga forest	- Mixed forest (larch, birch, pine)
	- Grasslands	- Alpine tundra	- Palsa landscapes (moss, lichens)
	- Peatlands (*Sphagnum magellanicum*)	- Boreal tussock tundra	- Thermokarst bogs (*Sphagnum* and *Eriophorum*)
		- Wetlands, including bogs and fens	
Lakes	- Peatland lakes	Mixotrophic and oligotrophic lakes formed from either Yedoma- or non-Yedoma permafrost soil	Mostly of glacial origin, influenced by permafrost degradation
	- Reservoir		
	- Glacial lakes		

In Alaska, the studied area ranged from the Alaska Range and Fairbanks area (interior, continental climate, 63–65°N, discontinuous permafrost) up to Toolik Field Station (North Slope, arctic climate, 66–69°N, continuous permafrost; Fig. 2). The physiochemistry and CH_4_ emissions of lakes ALL1 (Killarney lake), ALL2 (Otto lake), ALL3 (Nutella lake), and ALL4 (Goldstream lake) were previously characterized^35^. A number of heterogeneous soil and wetland samples were collected around the studied Alaskan lakes and/or from monitored sites, as detailed in Supplementary Table S1. In the Alaska Range and Fairbanks area, soils were mostly covered by mixed or taiga forests, alpine tundra, and bogs or fens wetlands. In the norther Brooks Ranges mountain system, the landscape was piedmont hills with a predominant soil of porous organic peat underlain by silt and glacial till, all in a permafrost state, characterized mainly by *Sphagnum* and *Eriophorum* vegetation, as well as dwarf shrubs.

In Siberia, the studied area was located in the discontinuous permafrost region surrounding Igarka, on the eastern bank of the Yenisei River (Fig. 2). This region was mainly covered by forest, dominated by larch (*Larix Siberica*), birch (*Betula Pendula*), and Siberian pine (*Pinus Siberica*), and palsa landscapes (frozen peat mounts), the latter being dominated by moss, lichens, Labrador tea and dwarf birch. In degraded areas, thermokarst bogs were dominated by *Sphagnum* spp. and *Eriophorum* spp. Land cover was an indicator of permafrost status, since forested areas reflected a deep permafrost table (>2 m) associated with Pleistocene permafrost, while palsa-dominated landscapes were indicative of the presence of near-surface (<1 m) Holocene permafrost. In this area, most of the lakes were of glacial origin and influenced by permafrost degradation^43^ that has been observed for the last 30 years, while some were thermokarst lakes (Supplementary Table S1). Two studies that focused on methane cycling in SIL1 to SIL4 were recently published^18,20^. We sampled organic soils on a degradation gradient from dry palsa to thermokarst bogs^44^, as detailed in Supplementary Table S1.

Subantarctic sites were located in three areas in the Southern part of Chilean Patagonia: the Magellanic region around Punta Arenas, Tierra del Fuego, and Navarino Island (Fig. 2). Most of the sampled lakes from Magellanic and Tierra del Fuego regions were of glacial origin, while Navarino Island lakes were peatland lakes, surrounded by peatland and broadleaf forests. Peatlands were characterized by a very low diversity of *Sphagnum* species dominated by *S. magellanicum* from hollows up to hummocks. The typical broadleaf forests of the area were dominated by *Nothofagus*. Some grassland soil came from an experimental monitored field site (Supplementary Table S1). Samples collected from Patagonian soils and wetlands have been included in a recent survey of soil geochemical characterization (organic content)^45^. Sediment samples collected in lakes PPL1, PPL2, PCL1, PCL2, PCP2 were also included in a recent study by Lavergne *et al*.^46^ which showed that increasing air temperature led to enhanced CH_4_ production and to an associated metabolic shift in the CH_4_ production pathway, increasing the relative contribution of hydrogenotrophic methanogenesis compared to acetoclastic methanogenesis, together with consistent microbial community changes.

Surface area for lakes and elevation for all sites were determined using Google Earth Pro. Climate variables (Table 1) for each site were retrieved from WorldClim – Global^47^.

### Sampling design

**Fig. 4:**
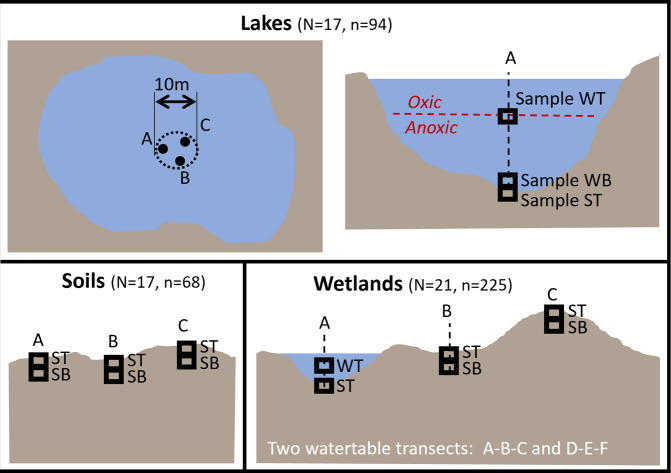
Sampling strategy for lake, soil and wetland sites (top, bottom left and bottom right panels, respectively). In lakes, at replicate points A, B and C, the water sample ‘WT’ was taken at the oxycline, and the water sample ‘WB’ just above sediment interface. One sediment sample was also collected. At soil sites, at replicate points A, B and C, two soil layers were sampled: ‘ST’ and ‘SB’ samples, representing respectively the top and bottom layers. In wetlands, two replicate transects were defined along the microtopography continuum hollow-edge-hummock. In hollows, one water and one solid sample were collected. At edges and hummocks, the same strategy as for soil sites was followed. For each type of sites, the number of sites and the corresponding number of events (*in situ* measurement and/or sampling) is indicated between parentheses.

A specific sampling strategy was defined for each kind of ecosystem, i.e. lakes, soils, and wetlands (Fig. 4), as follows.

In lakes, surface (0–10 cm) sediments and water samples were collected from three replicate points A, B, and C (Fig. 4) corresponding to the deepest zone of the lake, at ~ 2–5 meters of distance from each other. Two sampling depths were considered for the water samples: (i) at the oxycline, and (ii) just above the interface with sediment. Water was sampled using a 2.2 L Van Dorn bottle (Wildco, Mexico). Sediments were sampled using an Ekman dredge.

Mineral soil samples were collected from three replicate points A, B, and C (at ~ 2–5 meters of distance from each other), considering two sampling depths for each point (Fig. 4).

In wetlands, microtopography is known to influence organic matter decomposition, CH_4_ emissions, microbial community structure, and metabolic pathways^48–50^. The sampling strategy covered the three main microtopographic features of wetlands: hollows (i.e. small depressions, ponds, that can be filled with water or not at the time of sampling) (points A and D, Fig. 4); flat edges (or lawns) at the water table level or below, usually water-saturated and characterized by *Sphagnum* moss vegetation (points B and E, Fig. 4); and hummocks (i.e. dryer elevated mounts/raised domes, above the water table level, usually characterized by lichens and shrubs) (points C and F, Fig. 4). Two duplicate transects were considered, i.e. A-B-C and D-E-F transects, collected at ~ 10 meters of distance from each other. At each point, two sampling depths were considered, according to the same strategy as explained below for soils.

For both mineral and organic soils, soil blocks (20 × 20 × 20 cm blocks) were collected with a bread knife or a shovel. If soil layers could be clearly identified, top and bottom samples were defined accordingly and reported in the database. Otherwise, default depths were 0–10 cm for the surface layer and 10–20 cm for the bottom layer.

In addition to ecosystem-scale descriptors, every sample was characterized by point-scale descriptors (latitude, longitude, microtopography and vegetation type) and sample-scale descriptors such as environmental material (water, sediment, organic or mineral soil; Table 1 and Fig. 3). Soil samples were classified between organic and mineral soils using organic matter content (40% threshold) as the discriminating criterion between the two environmental materials^6^.

The material and methods used for characterizing these samples *in situ* and in the laboratory are described in the following sections. In some sites (ALP3, ALS3, ALS4, ALS6, ALS8, ALS9, PCL3, PCP3, PCS1, PPL3, PPP3, PTL1, PTL2, PTP1, PTP2, PTS1, SIL5, SIP6, SIP7, SIS3, SIS4), a basic characterization was carried out, due to harsh conditions and limited access. This basic characterisation included restrained set of measured parameters as listed in Table 1, yet enabling to fully fill the objective of this project. All the other sites were fully characterized, including the whole set of measured parameters as listed in Table 1, according to the environmental package (water, sediment, soil).

### *In situ* analyses

#### Physicochemical analyses

At each sampling point and depth in lakes and hollows, dissolved oxygen, temperature, pH, conductivity, and redox potential were measured in water with a multiparametric probe (HI 9828, Hanna Instrument, Mexico). The detection limits for dissolved O_2_ was 10 µg L^−1^. In soil ecosystems, temperature was measured with an insertion thermometer (Isolab, Laborgerate GmbH).

#### Dissolved CH_4_ and CO_2_ concentrations

In lakes, the dissolved CH_4_ and CO_2_ concentrations were measured at each replicated sampling point and depth with the membrane-integrated cavity output spectrometry method using an ultraportable greenhouse gas analyzer (UGGA, Los Gatos Research, USA)^51^. The detection limits for dissolved CH_4_ and CO_2_ concentrations were 5 nmol L^−1^ and 4 μmol L^−1^ respectively.

#### Atmospheric CH_4_ and CO_2_ emission rates

**Fig. 5:**
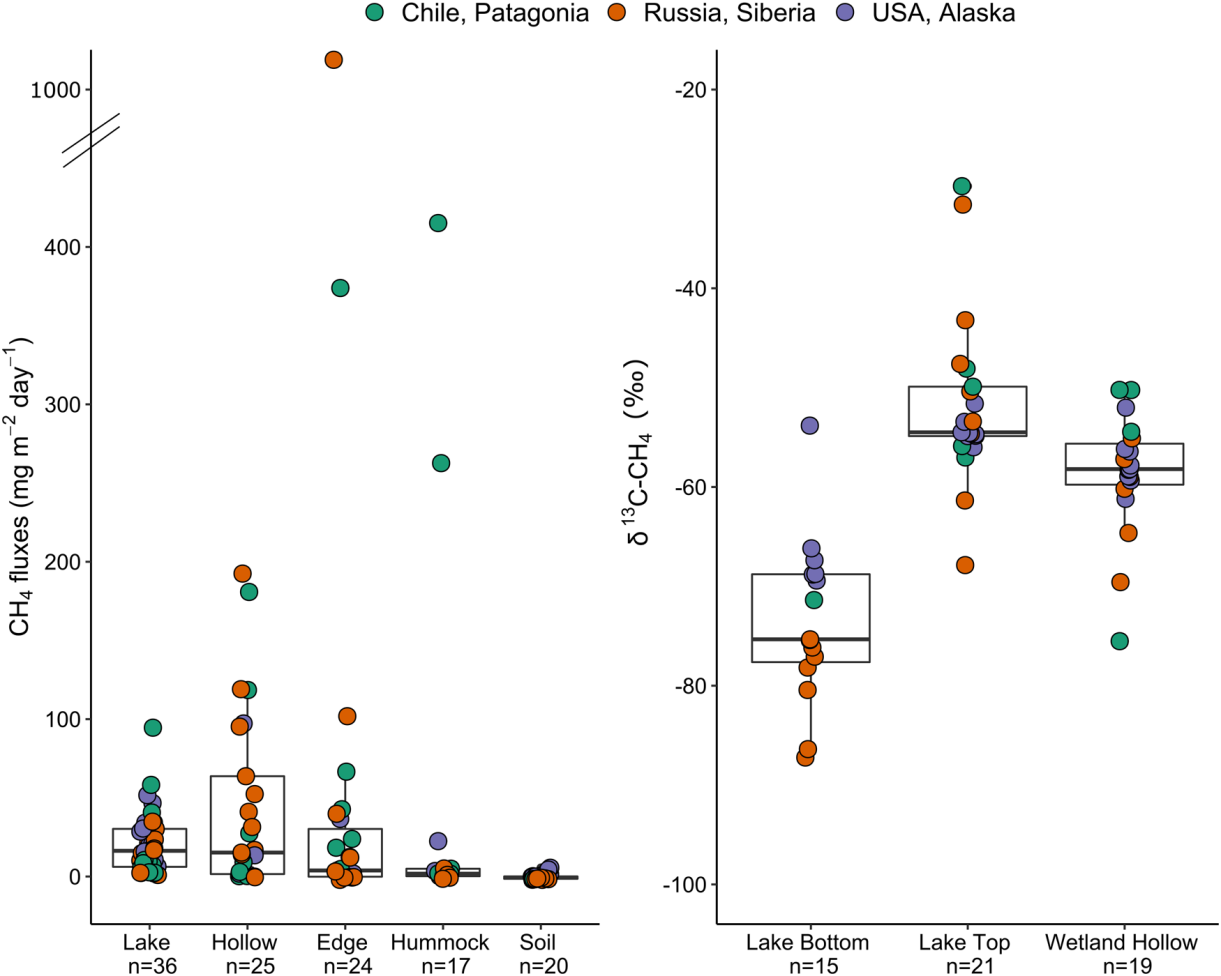
Methane emission rates measured during field campaigns (left) and δ^13^C-CH_4_ fractionation (right). Methane emission measurements using static chambers were pooled according to meaningful categories that combined the environmental feature, environmental package and microtopography descriptors. The δ^13^C-CH_4_ fractionation was measured in water samples only, i.e. in samples collected in the water column of lakes (at oxycline and at the bottom) and in hollows found in wetlands.

CH_4_ and CO_2_ emission rates were estimated with a static opaque chamber coupled in a loop to the UGGA (Los Gatos Research, USA), following the procedure described previously^9^. Briefly, a 0.102 m^2^ floating chamber (7.8 L) was placed at the surface of lakes and ponds and a 0.035 m^2^ chamber (12.3 L) was installed on soil sites. Accumulation of CH_4_ and CO_2_ was recorded during 5 min, and flux determined from the slope of CH_4_ and CO_2_. Then the chamber was ventilated and closed to perform another flux measurement. At least three replicate measurements were performed at each location (sampling points defined in Fig. 4). The static chamber method used measures the total flux at the surface, i.e. including both diffusive and ebullitive fluxes. As illustrated in Fig. 5a, the highest CH_4_ emission rates were found in hollows, especially in Siberian peatlands of discontinuous permafrost and lakes.

### Sample processing in the field

For further analysis, water subsamples were collected into 10 mL glass vials, directly in the field. For δ^13^CCH_4_, δ^2^HCH_4_, and total organic carbon (TOC) analysis, samples were acidified (HCl 6 N). For dissolved inorganic carbon (DIC) concentration and δ^13^C-DIC analysis, HgCl_2_ was added to the samples to stop any biological activity. After fixation by HCl and/orHgCl_2_, water subsamples were stored at 4 °C in dark conditions. Soil samples were also kept at 4 °C for 24 h maximum before further processing.

### Laboratory methods

#### Moisture and organic matter content

Soil and sediment samples were dried at 110 °C overnight to determine the dry weight. Organic matter content was assessed via loss on ignition at 550 °C.

#### Suspended solids

Lake and hollow water samples (20 mL to 3 L, until clogging) were filtered on pre-weighted combusted GF/F grade glass microfiber filters (0.7 µm pore size, Whatman). The filters were dried overnight at 105 °C to calculate the total suspended solids (TSS). The filters were then incinerated at 550 °C for 2 hrs to determine the concentration of particulate organic matter (POM).

#### Filtration

After pre-filtration at 80 µm (nylon net filters, Merck Millipore, Cork Ireland), water samples were filtered at 0.22 µm (nitrocellulose GSWP membrane filters, Merck Millipore, Cork Ireland) up to filter clogging (corresponding to 636 ± 521 mL on average, ranging from 70 to 2930 mL depending on the highly variable suspended matter content of the samples). The filter was frozen at −20 °C prior to DNA extraction. The filtrate was recovered and used to prepare four vials for further analysis of dissolved organic carbon (DOC), the isotopic composition (δ^13^C) of DOC, optical properties of dissolved organic matter, cations, anions, and trace elements.

#### Pore water extraction

The water extraction was carried out on soil and sediment samples to assess the mobile fraction of DOC, major anions and cations, trace elements, and the optical properties of dissolved organic matter (DOM). Following the procedure recommended in Jones & Willet^52^, 40 g of sample were placed in 200 mL of deionized water, and gently agitated with a magnetic stirrer at room temperature for 1 hr. The liquid phase was then recovered using a microRhizon sampler (Rhizosphere, Netherlands). The same procedure as for water samples was used to prepare and analyse these extracts.

#### Total and dissolved organic carbon

In water samples collected in lakes and hollows, TOC and DOC concentrations were analysed in using a TOC-V CSH analyser (Shimadzu, Japan). For DOC concentrations, samples were acidified to pH 2 using HCl 6 N and stored in 10 mL baked clear glass vials. The limit of quantification (LoQ) was 1 mg L^−1^.

#### Anions and cations

Major ions were quantified in water samples collected in lakes and hollows and in pore water using a HPLC (Dionex, USA), a Dionex DX-120 analyser for cations (Thermo Fisher Scientific, France) and a Dionex ICS-5000 + analyser for anions (Thermo Fisher Scientific, France), according to recommandations^53^. The LoQ was 0.5 mg L^−1^ for calcium, chloride, sulphate, and magnesium; 0.25 mg L^−1^ for bromide, sodium, and potassium; 0.025 mg L^−1^ for ammonium and phosphate; 0.01 mg L^−1^ for fluoride, nitrate, and nitrite.

#### Trace elements

For trace element analysis, samples were acidified with ultrapure HNO_3_ prior to ICP-MS (7500ce, Agilent Technologies) analysis, and kept in 15 mL polypropylene vials. LoQ were <0.5 µg g^−1^ for aluminium, iron, manganese, <0.05 µg g^−1^ for vanadium, chromium, cobalt, nickel, copper, zinc, and <0.005 µg g^−1^ for arsenic, strontium, cadmium, antimony, lead, uranium.

#### Optical properties

Subsamples were collected in 30-mL polypropylene vial for optical properties of DOM. The UV absorption spectra of pore water were measured with a spectrophotometer (Secoman UVi-lightXT5) from 190 to 700 nm in a 1 cm quartz cell. The specific UV absorbance at 254 nm (SUVA, L mg C^−1^ m^−1^) was calculated as follows: SUVA = A_254_/b*DOC^54^, where A_254_ is the sample absorbance at 254 nm (non-dimensional), b is the optical path length (m), and DOC is in mg L^−1^. Fluorescence measurements were performed using a spectrofluorometer (Synergy MX, Biotek). The emission spectrum was recorded for a 370 nm excitation wavelength. The fluorescence Index (FI) was determined for a 370 nm excitation wavelength, as the ratio of the 470 nm emission to 520 nm emission^55,56^.

#### Isotopes

The stable isotopic signature of methane (δ^13^C-CH_4_, shown in Fig. 5b, and δ^2^H-CH_4_) was analyzed at the Stable Isotope Facility of UC-Davis (https://stableisotopefacility.ucdavis.edu/methane-ch4-gas), using a ThermoScientific Precon concentration unit interfaced to a ThermoScientific Delta V Plus isotope ratio mass spectrometer (ThermoScientific, Germany). Methane was extracted for IRMS analysis following the method of Yarnes *et al*.^57^. The LoQ was 5 ppm of CH_4_ for δ^2^H and 1.7 ppm of CH_4_ for δ^13^C, and standard deviation was typically 2‰ for δ^2^H and 0.2‰ for δ^13^C. The δ^13^C-CO_2_ was analyzed using a mass spectrometer (Isoprime 100, Elementar, UK) coupled with an equilibration system (MultiFlow-Geo, Elementar, UK). Samples were acidified using phosphoric acid and flushed with helium. The δ^13^C-DOC was analysed at the UC Davis Stable Isotope Facility, following the described procedure (http://stableisotopefacility.ucdavis.edu/doc.html). A TOC Analyzer (OI Analytical, College Station, TX) was interfaced to a PDZ Europa 20–20 isotope ratio mass spectrometer (Sercon Ltd., UK) utilizing a GD-100 Gas Trap Interface (Graden Instruments).

#### DNA extraction

Soil and sediments were subsampled and frozen at −20 °C. DNA was extracted from 0.5 g of the soil or sediment subsamples and from the previously frozen 0.22-µm filters using the PowerSoil and PowerWater DNA isolation kits, respectively (Qiagen, Hilden, Germany), following manufacturer instructions. The DNA extracts were stored at −20 °C.

#### qPCR assay

**Fig. 6:**
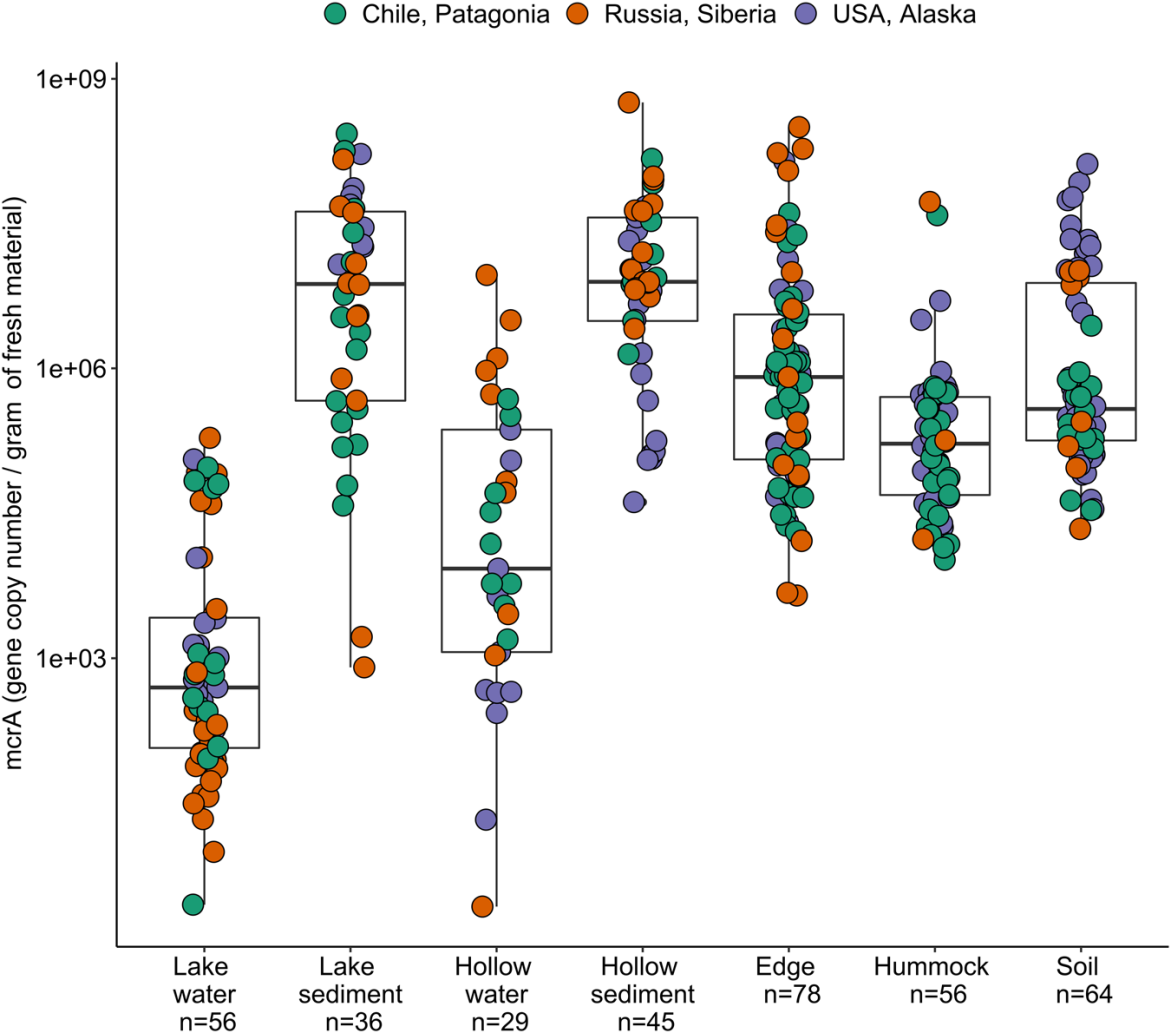
Abundance of methanogens in samples collected in Patagonia, Siberia and Alaska. Methanogen abundances were derived from qPCR assays targeting mcrA gene and were pooled according to environmental feature, environmental package and microtopography descriptors.

The abundances of four genes were measured by quantitative PCR (qPCR): bacterial 16S rRNA gene, archaeal 16S rRNA gene, *pmoA* gene (marker gene for aerobic methane oxidizing bacteria through the particulate methane monooxygenase), and *mcrA* gene (marker gene for methanogens and ANMEs through the methyl coenzyme M reductase). Duplicate measurements were run in 20 µL, using the Takyon SYBR master mix (Eurogentec, Belgium) with a CFX96 thermocycler (Bio-Rad Laboratories, Hercules, CA, US) and AriaMX thermocycler (Agilent, CA, US). Primer sequences and concentrations, thermocycling conditions, and standard curve preparation were detailed in Thalasso *et al*.^18^. As an illustration, the abundance of *mcrA* gene according to habitat (i.e. category combining the environmental material and the microtopography) is displayed in Fig. 6.

#### High-throughput amplicon sequencing

**Fig. 7:**
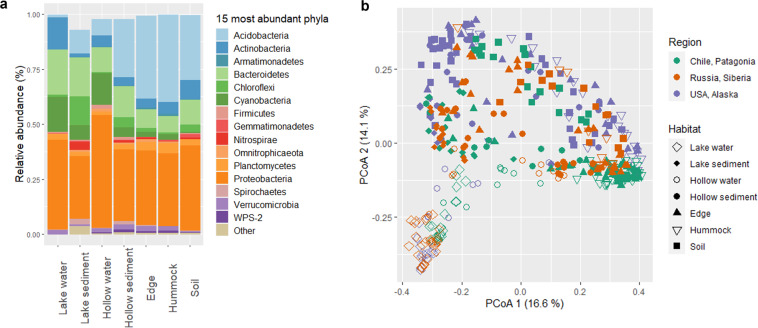
Taxonomic composition and similarities between the 387 microbial communities. The taxonomic composition of bacteria is presented at the phylum level, representing only the 15 most abundant phyla (panel **a**). Relative abundances of the phyla were calculated for seven habitats, i.e. a combination of environmental feature, environmental package and microtopography descriptors. Only the 15 more abundant phyla are displayed. The principal coordinate analysis (PCoA) of the filtered and standardized OTU abundance table was computed with Bray-Curtis distance to visualize similarities between microbial communities of the different habitats (indicated by the symbol shape) across the three studied regions (indicated by the symbol color; panel **b**). The percentage of total variance explained by each component is indicated along the axis, showing high microbial community variability mainly according to the different habitats.

Archaeal and bacterial diversity was assessed using metabarcoding and targeting the V4-V5 region of 16S rRNA gene. Amplicons were obtained from DNA extracts using 515 F (GTGYCAGCMGCCGCGGTA) and 928 R (CCCCGYCAATTCMTTTRAGT) primers^58^. MTP Taq DNA polymerase was acquired from Sigma (France). The thermocycling procedure was the following: 2 min at 94 °C; 30 cycles of 60 s at 94 °C, 40 s at 65 °C, and 30 s at 72 °C; and finally, 10 min at 72 °C. PCR products were used for pair-end sequencing using Illumina Miseq (2 × 250-bp). After pre-processing of raw reads through the FROGS pipeline^59^, a total of 18 369 310 sequences were obtained from the 387 samples, and clustered into 121 971 OTUs using Swarm^60^. The OTUs were further filtered at 0.005% of relative abundance, as previously recommended^61^, and taxonomically annotated against SILVA 132 rRNA database. Community analysis was carried out in R software, version 4.1.1, with ‘*phyloseq’* package^62^. The taxonomic composition of bacteria according to habitat was represented by a barplot at the phylum level (Fig. 7a). As an illustration of the microbial diversity outcomes from this dataset and the community variability according to the different habitats, the dissimilarity among the 387 community structures was visualized by a principal coordinate analysis PCoA, a.k.a. Multidimensional scaling (MDS) with the *ordinate* function using Bray Curtis distance (‘phyloseq’ package^62^) computed on the filtered and standardized (percentage) OTUs relative abundances (Fig. 7b).

## Data Records

This paper presents a combination of sample metadata, environmental data (gas flux and biogeochemical measurements), and high-throughput microbiome sequencing data co-located in time and space. Linking these data of different nature is crucial for their effective interpretation and reuse. The geo-referenced dataset was documented in the DRYAD platform^63^ and is fully available under a CC0 1.0 Universal (CC0 1.0) Public Domain Dedication license. The dataset is publicly accessible with the following doi: 10.5061/dryad.rfj6q57dp. The dataset in DRYAD also includes a ‘readme’ file intended to provide key information for understanding and reuse of the dataset. The data is organized in a standard datasheet table easily downloadable (csv format) with 387 samples (in rows) and 120 parameters (in columns). The first row of the table is the parameter name and the second row of the table is the unit of each parameter. The parameters are organized as follows: Sampling context; Ecosystem characteristics; Sequencing method details; Basic physicochemical parameters; Organic matter characteristics; Nutrients, anions, cations; Greenhouse gases; qPCR quantifications; Micro elements; Bioclimatic variables. The data is easily downloadable in csv format, and the clear and unique sample ID enables to link the data to the sequence set. The raw sequence data in FASTQ format without preprocess, were archived in the European Nucleotide Archive (ENA) with accession codes PRJEB36731 (Siberia)^64^, PRJEB36732 (Alaska)^65^, and PRJEB36733 (Patagonia)^66^. These microbial datasets along with sample metadata have been published in the Global Biodiversity Information Facility (GBIF)^67–69^ separately for Patagonia^67^, Siberia^68^, and Alaska^69^. Standardized information about sequence data^70^ were reported together with environmental data, and formatted as defined by the Genomic Standards Consortium^71^, based on MIMARKS sheet for miscellaneous natural environment.

## Technical Validation

Operator training and strategic harmonization for meta(data) collection occurred at the beginning of the first field campaign to ensure all operators used identical and replicable methods in terms of data acquisition in field, sampling, sample processing in the field and in the laboratory, and data recording. All data were checked and accurately transferred to MIMARKS database. The database was eventually manually curated by a dedicated data manager.

During CH_4_ and CO_2_ flux measurement, two criteria were tested before emission trends were validated^72^: (i) that the initial concentration was nearly equal to ambient atmospheric concentration; and (ii) that the linear correlation coefficient (R^2^) from the regression analysis reached 0.90. When a measurement did not meet these criteria, additional replicates were done, which occurred in only a few occasions.

Reference material ION-915 and ION 96.4, both acquired from Environment and Climate Change Canada (Canada), were included in the analytical loop of TOC and major anions and cations determination. Recovery was >95% of the certified value. The trace element certified river water^53^ SLRS6 (National Research Council – Conseil National de Recherches Canada) was used as a reference material on every run for ICPMS analysis, with indium as an internal standard, and accuracy (i.e. recovery >95%) was checked. The analytical routine included the analysis of blanks, calibration standards, and a multi-element quality control solution (EPOND) every 12 samples.

For isotopic analysis of δ^13^C-CO_2_ analysis, standards included Na_2_CO_3_ and NaHCO_3_ as well as internal water standards, that were analyzed every 8 samples to check for instrument stability. All samples were analyzed in replicates. Standard deviation was typically around or below 0.2‰.

Blanks (sterile pure water) were included in the DNA extraction process, PCR, and qPCR protocols. The absence of amplification on negative controls (contamination) was checked by gel electrophoresis. The correct size of 16S rRNA amplicons and the PCR specificity (unique band) were also checked by gel electrophoresis.

For qPCR of bacterial and archaeal 16S rRNA gene and *mcrA* gene, standard curves were prepared from 10-fold serial dilutions of each target gene, amplified from the following pure strains: *Pseudomonas stutzeri* SLG510A3-8 (KT153610 accession number), Arch_21F_10-Berre_sed clone (KT351355 accession number), and *Methanosarcina barkeri* CM1 (AKJ39604 accession number), respectively, and cloned in pGEM-T plasmid (Promega). For *pmoA*, the standard was synthetized by Eurofins from *Methylobacter* sp. BB5.1 *pmoA* gene sequence (AF016982 accession number), inserted in TOPO-TA pCR2.1 plasmid. qPCR efficiencies were always >90% and amplicon size and specificity were confirmed by melting curve analysis and agarose gels.

Two samples with less than 1325 sequences retrieved from high-throughput sequencing were discarded from the sequence datasets deposited in the European Nucleotide Archive.

## Supplementary information

Supplementary Table S1.

## Data Availability

No custom code was used to process the dataset presented in this article. To visualize microbial structure and composition, the diversity data provided in this dataset have been processed through the FROGS pipeline^59^ version 3.2.3 (http://frogs.toulouse.inra.fr/), available on the Galaxy server (https://vm-galaxy-prod.toulouse.inrae.fr/galaxy_main/) of the Genotoul bioinformatic platform (http://bioinfo.genotoul.fr/) under GNU GPLv3 licence.
