# Supplementary material for: A combined microbial and biogeochemical dataset from high-latitude ecosystems with respect to methane cycle
Source: Sci Data. 2022 Nov 4;9:674. doi: 10.1038/s41597-022-01759-8 (PMC9636175; doi:10.1038/s41597-022-01759-8)
Supplement: Supplementary file 1 — Supplementary Table S1. [file 41597_2022_1759_MOESM1_ESM.pdf]

Supplementary material for

Data Descriptor

A combined microbial and biogeochemical dataset from high-latitude ecosystems with respect to methane cycle

Maialen Barret[1], Laure Gandois[1], Frederic Thalasso[2], Karla Martinez Cruz[3,4], Armando Sepulveda Jauregui[3,5], Céline Lavergne[6], Roman Teisserenc[1], Polette Aguilar[6], Oscar Gerardo-Nieto[2,7], Claudia Etchebehere[8], Bruna Martins Dellagnezze[8], Patricia Bovio-Winkler[8], Gilberto J. Fochesatto[9], Nikita Tananaev[10,11], Mette M. Svenning[12], Christophe Seppey[12, 13], Alexander Tveit[12], Rolando Chamy[14], María Soledad Astorga-España[3], Andrés Mansilla[3], Anton Van de Putte[15], Maxime Sweetlove[15], Alison E. Murray[16], Léa Cabrol[17, 18]

List of content:

Supplementary Table S1. Main ecosystem characteristics of sampling sites in Patagonia (A), Alaska (B) and Siberia (C).

Supplementary Table S1. Main ecosystem characteristics of sampling sites in Patagonia (A), Alaska (B) and Siberia (C).

A. Patagonia sites.

Sampling site name	Regional sector	Main ecosystem characteristics
PCL1 (Castor lake)[a]	Navarino Island	Peatland lake, surrounded by peatland and broadleaf forest
PCL2 [a]	Navarino Island	Peatland lake, surrounded by peatland and broadleaf forest
PCL3 (Zanartu lake)	Navarino Island	Wetland/pampa lake
PCP1	Navarino Island	*Sphagnum magellanicum* dominated peatland, adjacent to PCL1
PCP2 [a]	Navarino Island	Mejillones peatland, dominated by S. magellanicum
PCP3	Navarino Island	*S. magellanicum* dominated peatland, adjacent to PCL2
PCS1	Navarino Island	Broadleaf forest soil
PPL1 (Hambre Lake)[a]	Magellanic	Post-glacial lake, in a protected area (Puerto del Hambre) surrounded by *Nothofagus* forest
PPL2 (Lynch lake)[a]	Magellanic	Natural lake converted into a reservoir, slightly urbanized area, surrounded by *Nothofagus* forest and grass steppe
PPL3 (Cabo Negro lake)	Magellanic	Steppe landcape, Magellanic strait
PPP1	Magellanic	Peatland in the National Reserve of the Parillar lake
PPP2	Magellanic	San Juan bog, edge of PPL1
PPP3	Magellanic	Peatland south of Puerto Natales, Ultima Esperanza province
PPS1	Magellanic	Typical broadleaf forest soil of the area, dominated by *Nothofagus,* edge of PPL2
PPS2	Magellanic	Grassland soil from an experimental field site [b]
PTL1 (Los Cisnes lake)	Tierra del Fuego	Steppe landcape, Porvenir sector, Natural protected area, stromatolithes
PTL2 (Blanco Lake)	Tierra del Fuego	In Karukinka National Park, surrounded by steppe, forest and peatlands
PTP1	Tierra del Fuego	*S. magellanicum* dominated peatland
PTP2	Tierra del Fuego	*S. magellanicum* dominated peatland, adjacent to PTL2
PTS1	Tierra del Fuego	Grassland

[a] Methane production from sediment samples of these lakes was previously characterized[46]

[b] monitored by the Chilean Institute of Agriculture and Livestock Research (INIA)

B. Alaska sites.

Sampling site name	Regional sector	Main ecosystem characteristics
ALL1 (Killarney lake)[a]	Fairbanks	Mixotrophic, formed from Yedoma-type permafrost soil, in aeolian sedimentary deposits, surrounded by northern boreal forest
ALL2 (Otto lake)[a]	Alaska Range	Oligotrophic, formed from non-Yedoma permafrost soil, surrounded by forest tundra
ALL3 (Nutella lake)[a]	Alaska Range	Oligotrophic, formed from non-Yedoma permafrost soil, surrounded by alpine tundra
ALL4 (Goldstream lake)[a]	Fairbanks	Mixotrophic, formed from Yedoma-type permafrost soil, in aeolian sedimentary deposits, surrounded by northern boreal forest
ALP1	Fairbanks	Wetland at the edge of ALL1
ALP2	Alaska Range	Wetland in tundra landscape
ALP3	Alaska Range	Wetland around ALL3
ALP4	Fairbanks	Peat from the taiga forest bog in the Poker Flat Research Range (UAF) in Chatanika, characterized by discontinuous permafrost [b]
ALP5	Norther Brooks Range	Wet sedge from riparian fen at the UAF-Toolik Lake Field Station site
ALP6	Norther Brooks Range	Fen soil from Inmavait Creek Basin Research Site (NSF-AON Arctic Observing Network)
ALS1	Fairbanks	Mixed forest soil at the edge of ALL1
ALS2	Alaska Range	Coniferous forest soil
ALS3	Alaska Range	Wetland soil at the edge of ALL2
ALS4	Alaska Range	Coniferous forest soil
ALS5	Alaska Range	Tundra soil around ALL3
ALS6	Fairbanks	Subarctic alpine tundra soil in Pinnell Mountain
ALS7	Norther Brooks Range	Tussock tundra hillslope soil at the UAF-Toolik Lake Field Station site
ALS8	Brooks Range	Wetland on the south side of the Brooks Range
ALS9	Brooks Range	Bog soil on the south side of the Brooks Range

[a] The physiochemistry and CH_4 emissions from these lakes were previously characterized[35]

[b] LTER monitored site: www.lter.uaf.edu/research/study-sites-cpcrw

C. Siberia sites

Sample name	Regional sector	Main ecosystem characteristics
SIL1	Igarka	Lake of glacial origin, influenced by permafrost degradation
SIL2	Igarka	Lake of glacial origin, influenced by permafrost degradation
SIL3	Igarka	Lake of glacial origin, influenced by permafrost degradation
SIL4	Igarka	Lake of glacial origin, influenced by permafrost degradation
SIL5	Igarka	Thermokarst lake, belonging to the SIP6-SIP7 complex
SIP1	Igarka	Palsa dome of the SIP1-SIP2 complex, with small thermokarst bogs (ponds) perched on palsa, formed by recent thawing
SIP2	Igarka	Large wetland bog from the flat zone of the SIP1-SIP2 complex (adjacent to SIP1)
SIP3	Igarka	Small thermokarst bog developed in a mineral forest soil context (free from permafrost peatland)
SIP4	Igarka	Large wetland bog from the flat zone of the SIP4-SIP5 complex, crossing Fox Creek (adjacent to SIP5)
SIP5	Igarka	Palsa dome of the SIP4-SIP5 complex, with intermediate thermokarst bogs (ponds) formed by collapsing palsa
SIP6	Igarka	Palsa dome of the SIP6-SIP7 complex, with small thermokarst bogs (ponds) perched on palsa, formed by recent thawing
SIP7	Igarka	Large wetland fen from the flat zone of the SIP6-SIP7 complex, crossing the Gravyika river (adjacent to SIP6)
SIS1	Igarka	Mineral soil from typical mixed larch and birch forest, adjacent to SIP1
SIS2	Igarka	Mineral soil from typical mixed larch and birch forest, adjacent to SIP3
SIS3	Igarka	Mineral soil from typical mixed larch and birch forest, adjacent to SIL4
SIS4	Igarka	Mineral soil from typical mixed larch and birch forest, adjacent to SIL2
